# A Nanostructured SERS Switch Based on Molecular Beacon-Controlled Assembly of Gold Nanoparticles

**DOI:** 10.3390/nano6020024

**Published:** 2016-01-22

**Authors:** Yansheng Li, Yaya Cheng, Liping Xu, Hongwu Du, Peixun Zhang, Yongqiang Wen, Xueji Zhang

**Affiliations:** 1 Department of Chemistry and Biological Engineering, University of Science and Technology, Beijing 100083, China; shanshui4956@126.com (Y.L.); cy172872060@163.com (Y.C.); xuliping@ustb.edu.cn (L.X.); hongwudu@ustb.edu.cn (H.D.); zhangxueji@ustb.edu.cn (X.Z.); 2 Peking University People’s Hospital, Beijing 100083, China

**Keywords:** molecular beacon sensors, switch, self-assembly, surface-enhanced Raman scattering (SERS), gold nanoparticles

## Abstract

In this paper, highly purified and stable gold nanoparticle (AuNP) dimers connected at the two ends of DNA linkage were prepared by a versatile method. A nanostructured, surface-enhanced Raman scattering (SERS) switching sensor system was fabricated based on the controlled organization of gold nanoparticles (AuNPs) by a DNA nanomachine through the controlled formation/deformation of SERS “hotspots”. This strategy not only opens opportunities in the precise engineering of gap distances in gold-gold nanostructures in a highly controllable and reproducible fashion, but also provides a unique ability to research the origin of SERS and sequence-specific DNA detection.

## 1. Introduction

Surface-enhanced Raman scattering (SERS) is a technique that enhances Raman scattering by molecules adsorbed or modified on rough noble metal surfaces or nanostructures [1,2]. In comparison with the fluorescence spectrum, SERS shows a high sensitivity, has a much richer molecular structural information, is less prone to photobleaching, and especially has a greater degree of multiplexing capability [3,4,5]. With the advent of nanotechnology, many technologies have been proposed to produce efficient SERS-active systems in recent years [6,7,8,9,10,11,12]. Despite great progress, quantitative research on SERS origin and practical applications is often hampered due to the poor controllability and reproducibility in the substrate fabrications. Preparing a SERS substrate with higher stability, reproducibility and well-defined structure remains a challenge [6,8].

The rapid growth of DNA nanotechnology has provided a useful platform for controlling the aggregation of nanoparticles and constructing functional nanodevices due to its unique molecular recognition capabilities [13,14]. By flexible sequence design, researchers not only created desired static two-dimensional (2D) [15,16] or three-dimensional (3D) nanoarchitecture, but various motional DNA were also developed [17,18,19,20,21,22]. Examples include molecular beacons (MB) [18], DNA tweezers [19], hybridization-powered molecular motors [20], walkers and the aptamer-based switch [22]. Among them, molecular beacons (MB) are a kind of simple DNA nanomachine. Through binding-induced conformational changes that “open” and “close” the hairpin structure of MB, fluorescence-based detection, especially resonance energy transfer (FRET) between the quencher and fluorophore located at opposite ends of the MB, were commonly used for biomolecular recognition in biology, biotechnology and medical sciences.

In this paper, we have demonstrated a high-yield preparation strategy to obtain single-stranded DNA (ssDNA)-bridged AuNP dimers, where the stability of the AuNP-DNA conjugates was tremendously increased due to the polyvalent linkages between AuNPs and cyclic disulfide group-modified DNA. With this building block, a SERS structural switch was then constructed based on an elaborately designed molecular beacon structure. By the ordinal addition of two complementary fueling strands, the gap distances between two AuNPs could be reversibly controlled between a “shortened” (forming electromagnetic “hotspots” in SERS) and a “extended” state. With this SERS switch, the controllable and reproducible SERS enhancement was demonstrated through the controlled formation of SERS “hotspots”. This nanogap-engineering of AuNPs strategy not only provides new ways of overcoming long-standing problems in Raman and material research about controlling the nanometer gap, but provides a unique ability to detect specific DNA directly in aqueous environments, which is expected to benefit to research on the origin of SERS and further SERS applications.

## 2. Results and Discussion

It is well known that “hotspots” at the junctions between nanoparticles can give rise to large electromagnetic enhancements that enable SERS detection at or near single molecule sensitivity [5,6,7,8]. Despite considerable promise, until now, the methods for preparation of discrete nanoparticle dimers in high-purity and well-defined structures are relatively rare. The recent development of DNA nanotechnology has made it possible to engineer nanoparticles at nanometer-length scale [17,18,19,20,21,22,23,24,25,26]. In this research, highly purified DNA-AuNP conjugates with two AuNPs connected at the two ends of single-strand DNA (ssDNA) were firstly fabricated for the preparation of the SERS switch. In contrast to most cases of creating AuNP constructions by DNA assembly, where single alkane thiol modification on either the 3′ or 5′ end of the DNA was used to attach on AuNPs [13,17,22], in our strategy two molecular anchors, cyclic disulfide–containing phosphoramidite derivatives, were directly incorporated into the DNA molecular beacon sequence (60 bases) at the two ends as independent units utilizing standard phosphoramidite chemistry. It had been proved that cyclic disulfide can form strong polyvalent linkages with the surface of Au nanoparticles, which could increase the stability at higher salt concentration than the monothiol group [27].

**Figure 1:**
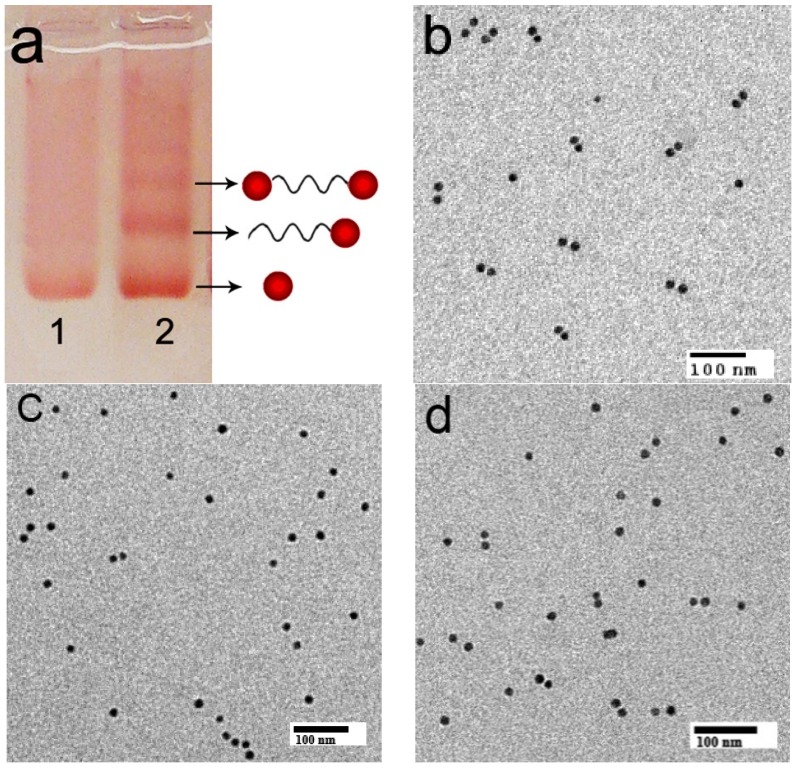
Characterization of discrete DNA-AuNP conjugates: (**a**) Electrophoretic analysis (2.5% agarose gel) of ssDNA-bridged AuNP dimers formed from molecular beacon sequences with 15 nm AuNPs. Lane 1: Bare AuNP, as a reference; Lane 2: A mixture of AuNP with the two cyclic disulfide–modified DNA strands. (**b**) Typical transmission electron microscopy (TEM) image of AuNP dimers separated by agarose gel electrophoresis. (**c**) Typical TEM image of AuNP-DNA separated by agarose gel electrophoresis. (**d**) Typical TEM image of AuNP dimers produced by the traditional hybridization method with two complementary AuNP-DNA conjugates.

The molecular beacon sequences MB-1 were mixed with excess AuNPs (15 nm) and incubated for 12 h. Agarose gel electrophoresis was used to separate AuNP dimers connected with a two-end-modified ssDNA from the product mixtures. As is known, agarose gel electrophoresis could be used to purify gold nanocrystals conjugated to controlled numbers of DNA strands. When DNA-gold conjugate structures are analyzed by agarose gel electrophoresis, they give discrete bands corresponding to specific numbers of gold nanoparticles with specific numbers of DNA strands. Compared to the separation method using high-performance liquid chromatography (HPLC), this method is particularly suitable to separate gold nanoparticles smaller than 15 nm and their conjugates with long-strand DNA. As shown in lane 2 of Figure 1a, the band displaying a slower mobility than bare AuNPs and single AuNP-DNA conjugates can be ascribed to the ssDNA-bridged AuNP dimers. Cutting and recovering this band from the gel according to the method in the literature, AuNP dimers could be obtained [17,28]. Polyvalent linkages were expected to be helpful to increase the stability of DNA-bridged AuNP dimers in contrast to that prepared with the conventional mercaptohexyl group monothiol [27,28,29]. The typical transmission electron microscopy (TEM) result further proved the dimer product had a high purity (Figure 1b). From statistics analysis of additional TEM fields of view obtained from the dimer band on the agarose gel, the AuNP dimers were produced in *ca.* 75% yield [28]. In contrast with the method of producing AuNP dimers with the traditional hybridization method with two complementary AuNP-DNA conjugates (Figure 1d, dimer percentage: *ca.* 54%), our method could obtain a higher AuNP dimer percentage. In this design, the role of the DNA is three-fold: connecting and confining the two AuNPs; controlling the distance between the two AuNPs through a deliberately designed hybridization process; being helpful in isolating and obtaining highly purified AuNP dimers. The AuNP surfaces of purified AuNP-DNA conjugates were further covalently labeled with a layer of Raman reporter species para-aminothiophenol (p-ATP) via a thiol group.

**Scheme 1:**
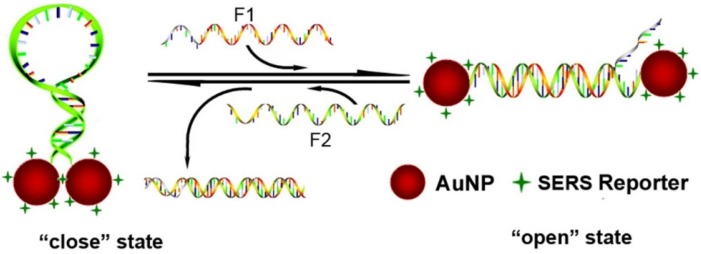
Schematic representations of surface-enhanced Raman scattering (SERS) switch through the control of the distance between the two AuNPs by sequential addition of fueling/analytes sequences of F1 and F2.

**Figure 2:**
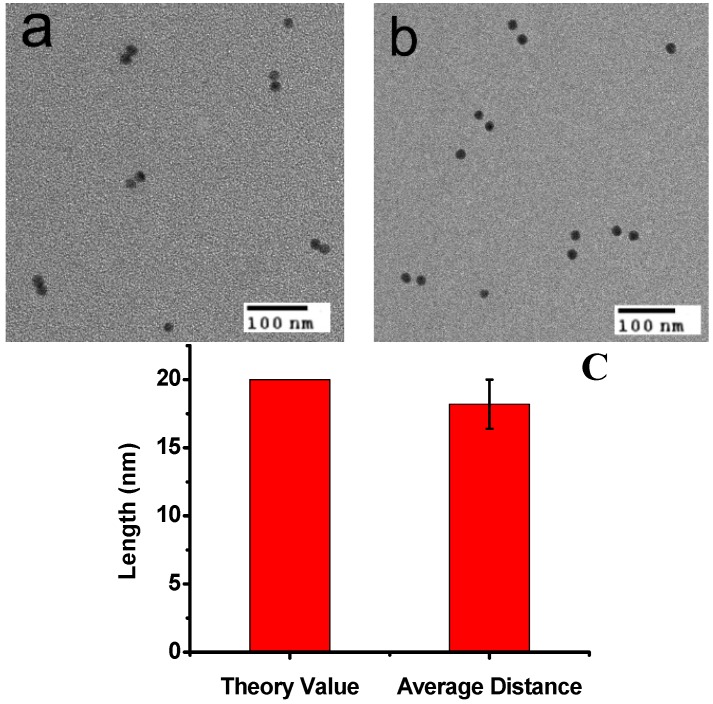
(**a**) TEM observation of AuNP dimers at “close” states and (**b**) AuNPs dimers at “open” states; (**c**) A histogram of the distance of the open form. The average distance was about 18.2 nm, which is coincident with the theoretical value for DNA 60 bases (~20 nm).

To realize the control and manipulation of AuNPs, a DNA sequence was designed into the MB switch. In this design, the DNA sequences were composed of one target-recognition region of 32 bases flanked by two short complementary stem sequences (nine-base pairs), which force the entire sequence to form a stem-loop structure in the absence of a target. The structure and operation principle of the switch are shown in Figure 1. In the absence of targets, two nine-base pairs at the ends of the sequence self-hybridize to form a stiff arm, thus pulling the two ends close together and bringing the two AuNPs into close proximity with each other (Scheme 1 “colse” state). The molecular beacons exist in a “close” state, and form the “hotspots” in SERS, as shown in the TEM images (Figure 2a).

In the presence of a fueling strand (or analyte) F1, which possesses a region that is fully complementary to the loop domain of the MB structure, hybridization between F1 and the loop sequence of MB takes place, and the stronger intermolecular hybridization opens the weaker stem helix, and further pushes the two arms apart into the straightened configuration (“open” state in Scheme 1), leading to the two AuNPs far away (about 18.2 nm). The spatial separation of the two AuNPs results in the disappearing of “hotspots” in SERS, as the TEM images show in Figure 2b. In the “open” state, a short single-stranded region remains in F1. When a fueling (or analyte) strand F2, which is fully complementary to F1, is added, it starts to hybridize at the dangling-end stretch of the straightened configuration. The strand F1 could be displaced via branch migration. Displacement of the strand F1 generates a waste double-stranded product and restores the device to its relaxed configuration, which further self-hybridizes to form the stable “close” configuration. Thus, the alternate addition of F1 and F2 strands can drive a cyclical operation of the device.

**Figure 3:**
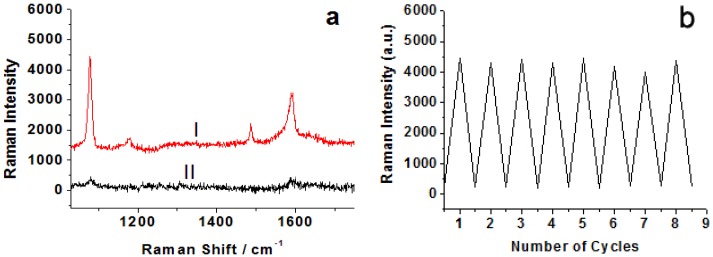
(**a**) SERS spectra of 4-aminothiophenol (ATP) molecules measured on nanostructured switch devices at “colse” (I) and “open” states (II), respectively. The spectra were recorded by using 633 nm laser lines. (**b**) A plot of the Raman intensity at 1078 cm^−1^ for 4-ATP molecules as a function of the number of cycles. High and low Raman absorbance maxima values indicate the devices at the “close” and the “open” states, respectively.

The large signal enhancements in the SERS mainly originate from electromagnetic field enhancement due to resonances of the optical fields with surface plasmons of noble metallic nanostructures. “Hotspots” at the junctions between nanoparticles can give rise to large enhancements that enable SERS detection at or near single molecule sensitivity. Based on the reversible structural transformation, this well-designed DNA device can realize the forming and deforming of the “hotspots” in SERS and lead to the switch “on” and “off” of Raman signals in a controlled manner. Figure 3a shows the comparison of SERS spectra of the p-ATP molecule on nanostructured switch devices at “close” and “open” states. At the “close” state, the Raman spectrum clearly displays two predominant Raman peaks at 1078 cm^−1^ and 1592 cm^−1^ (Figure 3a, curve I), which are associated with the C–S and C–C stretching vibrations, respectively [30], while at the open state, the Raman spectrum shows very weak SERS signals. The nanostructured DNA SERS devices can be switched between the straightened and closed states many times. To demonstrate the reversible switching of the nanoparticle aggregation, consecutive cycles were carried out between its straightened configuration and its close configuration by repeated sequential addition of fueling (or analyte) F1 and F2 strands. After each addition, Raman spectra of the samples were recorded and the strongest Raman band at 1078 cm^−1^ was plotted as a function of the number of cycles (Figure 3b). The analysis of the Raman absorbance maximum at 1078 cm^−1^ observed for the “close” and “open” states indicated a good stability in the intensity from cycle 1 to cycle 8. The results clearly demonstrate the feasibility of using fueling oligomers for the reversible switching of SERS.

**Figure 4:**
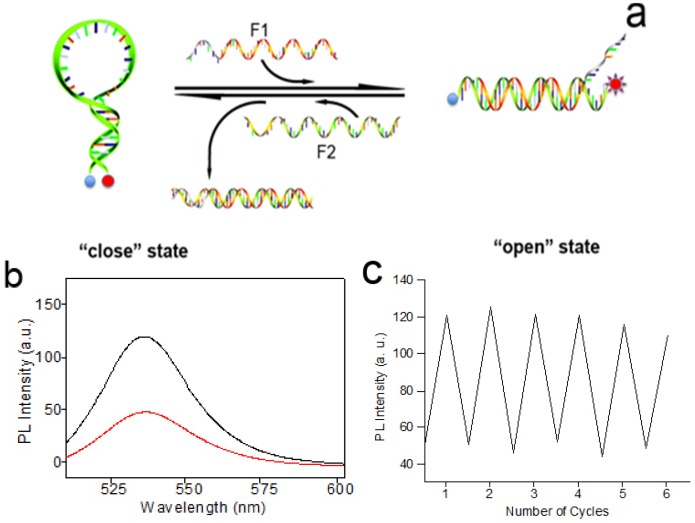
(**a**) Demonstration of the structural changes of the DNA nanomachine after the addition of F1 and F2. (**b**) Fluorescence spectroscopy after the addition of F1 (black curve) and F2 (red curve). (**c**) Fluorescence intensity showing the reversible switching of the DNA nanoswitch.

In order to further prove the switching effect, another DNA sequence MB-2 was prepared, and MB-1 and MB-2 have the same sequences. A quencher (dabcyl) and a fluorophores (rhodamine green) were labeled at the 5′ ends and the 3′ ends of the DNA MB-2, respectively. The same experimental conditions were used for this system as those used for the SERS experiment. Initially, DNA MB-2 formed the stem-loop structure in the absence of a target, which brought the F and Q into close proximity, resulting in fluorescence quenching. As depicted in Figure 4, in the presence of F1, the interaction between MB-2 and F1 destroyed the loop structure and the fluorescence intensity increased. The solution in the absence of F1 exhibited low fluorescence, while the same sample in the presence of F1 clearly displayed an increase in fluorescence intensity (Figure 4b). Moreover, the structures also showed the reversible switching from the changes of the fluorescence intensity (Figure 4c). This switching effect can also be used for sequence-specific DNA detection. When a specific target strand FT (corresponding to F1 or F2) is added, the AuNP aggregation or separation would effectively turn “on” or “off” the degree of electromagnetic enhancement of the reporter molecule, giving rise to a large “increase” or “decrease” in the SERS or fluorescence signals.

## 3. Experimental Section

### 3.1. Chemicals and Materials

Tris(hydroxymethyl)-aminomethane (Tris), boric acid, acetic acid, ethylenediamine-tetraacetic acid, NaCl, and MgCl_2_∙6H_2_O were purchased from Sigma-Aldrich (Shanghai, China). The 1,2-Dithiane-4-O-Dimethoxytrityl-5-[(2-cyanoethyl)-N,N-diisopropyl]-phosphora-midite (DTPA) was synthesized according to the literature procedures [28]. Microcon^®^ size-exclusion centrifugal filter devices were purchased from Millipore (Shanghai, China). Citrate-coated gold nanoparticles were made in-house. The ligand bis(p-sulfonatophenyl) phenylphosphine dihydrate dipotassium salt was purchased from Strem Chemicals (Kehl, Germany). Standard automated oligonucleotide solid-phase synthesis was performed by a Mermade 4 DNA/RNA synthesizer using conventional phosphoramidite chemistry.

The sequences are listed below:

MB-1 sequence: 5′-(DTPA) T CGA CGT AGTAT AGT GAC CAG TCG TAT C TTTT TTT GGA CCT GTC GTC TGTTC TAC GTC GA (DTPA)-3′

MB-2: 5′-(dabcyl)-T CGA CGT AGTAT AGT GAC CAG TCG TAT C TTTT TTT GGA CCT GTC GTC TGTTC TAC GTC GA-(rhodamine green)-3′

F1: 5′-AACAGACGACAGGTCCAAAAAAAGATACGACTGG TCACTATAATCAG GTC-3′

F2: 5′-GACCTGATTATAGTGACCAGTCGTATCTTTTTTTGG ACCTGTCGTCTG TT-3′

### 3.2. Experiment Procedure

Gold colloids were synthesized by the citrate/tannic acid method [7], and the mean diameter was 15 ± 1.2 nm. The citrate-stabilized gold colloids were then added with a negatively charged phosphine shell using bis(p-sulfonatophenyl) phenylphosphine dehydrate dipotassium salt to exchange the surface species [11]. DNA-AuNP conjugates were prepared by mixing AuNPs with the modified ssDNA with a mole ratio 3:1 and incubated for 12 h in 0.5× TBE buffer (containing 50 mM NaCl). DNA-AuNP monoconjugates and AuNP dimer were isolated from the reaction mixture by gel electrophoresis (2.5% agarose gel at 10 V/cm, 0.5× TBE buffer), followed by recovery of the appropriate band. The AuNP surfaces of purified AuNP-DNA conjugates were further covalently labeled with reporter species at 3 mM via a thiol group in buffer (0.5 × TBE, 50 mM NaCl) for 12 h. The AuNP surfaces of purified AuNP-DNA conjugates were further covalently labeled with reporter species via a thiol group in buffer (0.5× TBE, 50 mM NaCl) for 12 h. The modified AuNP-DNA conjugates were then resuspended in 0.5× TBE buffer (containing 100 mM NaCl) and further hybridized with equimolar quantities of complementary conjugates F1 to create AuNP “open” constructions (each 3.5 nM) overnight at 20 °C. For driving the switch, two equivalents of oligonucleotide F2 were added to the opened construction solutions, and the mixture was incubated at 20 °C for 2 h to reach the stable “colse” configuration state. Subsequently, the “colse” state was reversed to the “straightened” state by adding F1 (two equivalents with respect to F2). Repeated switching was performed by the alternate addition of two complementary fueling strands (or analyte).

### 3.3. Characterization

UV-Vis experiments were conducted on a Hitachi U4100 spectrophotometer (Tokyo, Japan). Transmission electron microscopy (TEM) images were obtained using a JEM-2100 200 kV electron microscope (Tokyo, Japan). Then 5.0 μL of dilute aqueous sample was spotted on the surface of Formvar-coated copper TEM grids. The sample was left on the grid for 3 min to allow particles to adsorb to the surface before touching the edge of the grid with a filter paper to wick off excess moisture and salts. Grids were then allowed to air-dry prior to analysis. Raman spectra were taken on a Renishaw invia Raman Microscope (London, UK). The spectra were recorded by using 633 nm laser lines.

## 4. Conclusions

In conclusion, although many efficient SERS analysis systems have been fabricated so far, most of them were based on nanostructured surfaces or large ensembles of nanoparticles. The controllability and reproducibility of SERS using such nanostructures were usually low, which hampers the progress of thorough investigations of SERS origin and SERS-based technological applications. In this manuscript, discrete and stable nanoparticle structures were elaborately fabricated. High-purity ssDNA-bridged AuNP dimers were prepared through incorporating two cyclic disulfide–containing phosphoramidite derivatives into the DNA sequence at the two ends. Based on this as-prepared DNA-AuNP conjugate, a nanostructured device was especially designed to serve as a SERS switch system, where the SERS “hotspot” structures can be formed or deformed with excellent reproducibility and controllability through the control of the distance between the two labeled AuNPs by the alternate addition of two complementary fueling strands. Sequence-specific DNA (*i.e.*, F1 and F2) detection could be realized on the basis of this SERS switch. Moreover, such a method could be further extended to other DNA sequence detection through the reasonable design of more DNA assembly structures. Such discrete and stable nanoparticle structures could potentially be further used as theoretical models in SERS mechanism research. Furthermore, the precise control of nanostructures in the nanometer size range might lead to widespread interest in SERS use for diagnostic applications and biological imaging, and it also has potential applications for other more important analytes such as specific DNA, proteins, enzymes, and drug molecules.
